# Aortic Dissection in a Case of Peripartum Cardiomyopathy

**DOI:** 10.1055/s-0041-1736672

**Published:** 2021-10-04

**Authors:** Tania Tabassum, Andrew Brazier, Miguel Garcia, Victoria Pettemerides, James Barnard

**Affiliations:** 1 Department of Cardiac Surgery, Manchester University National Health Service Foundation Trust, Wythenshawe Hospital, Manchester, United Kingdom; 2 Department of Cardiac Anaesthesia, Manchester University National Health Service Foundation Trust, Wythenshawe Hospital, Manchester, United Kingdom; 3 Department of Cardiology, Manchester University National Health Service Foundation Trust, Wythenshawe Hospital, Manchester, United Kingdom

**Keywords:** ECMO, peripartum cardiomyopathy, Type A aortic dissection, cardiomyopathy

## Abstract

Management of acute Type A aortic dissection can be complicated by patient comorbidities. We describe the case of a 29-year-old female with preexisting peripartum cardiomyopathy who developed a Type A dissection. Surgery was performed and venoarterial extracorporeal membrane oxygenation (ECMO) was instituted. She left hospital on the 71st postoperative day. It is extremely rare for a patient with cardiomyopathy to develop an aortic dissection. Deferring this patient's surgery to an ECMO center was crucial for her survival.

## Introduction

Type A aortic dissection is a phenomenon encountered by cardiothoracic surgeons across the world. Cases can vary in complexity and the perioperative planning for these patients is important in ensuring a positive outcome. Here we describe a case of acute Type A aortic dissection complicated by preexisting peripartum cardiomyopathy (PPCM). We could only find one example in the literature of these two conditions coexisting and presenting together. 1 In our case, the patient had an established diagnosed cardiomyopathy when she presented with an acute dissection. It is very rare for an aortic dissection to present in the presence of a patient with a cardiomyopathy. 2 PPCM is defined as systolic heart failure presenting within the last month of pregnancy or 5 months after delivery in the absence of any identifiable cause of heart failure. 3 Approximately two-thirds of these cases will present after delivery of the baby, usually within the first month. In the United States, an incidence of 10.3 per 10,000 live births is reported. 4 Aortic dissection is extremely rare, with an estimated incidence of 5 to 30 per 1 million people per year. 5 Although the etiology of this patient's dissection remains unclear, aortic dissection following pregnancy is a recognized phenomenon with approximately half of all aortic dissections in women under 45 years of age, being associated with pregnancy.

## Case Presentation



**Video 1** Preoperative transesophageal echocardiogram.


**Video 2** Transesophageal echocardiogram following recovery.


**Fig. 1:**
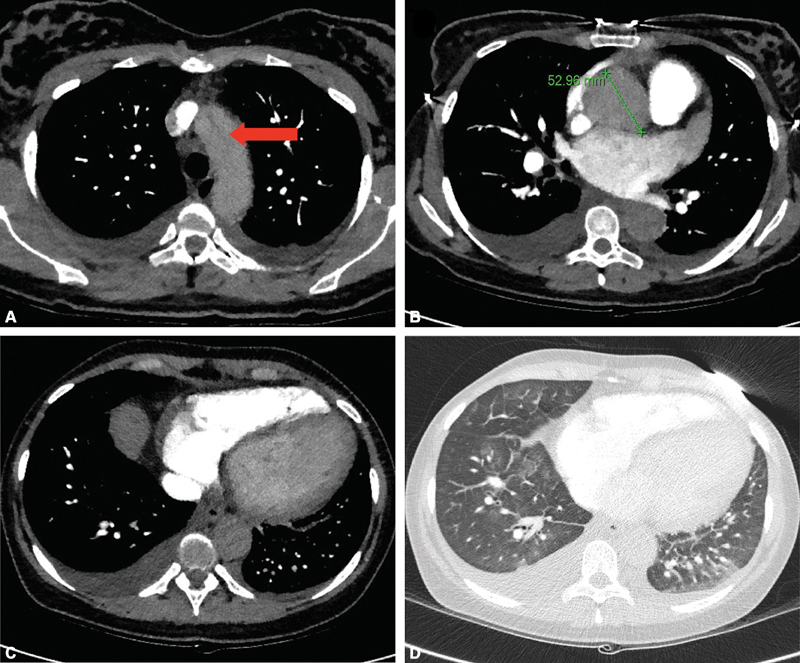
( **A** ) Axial image computed tomography pulmonary angiogram showing dissection flap (red arrow) and small bilateral pleural effusions. ( **B** ) Dilated aortic root (5.3 cm) although the phase of contrast (i.e., optimized for pulmonary artery opacification) does not allow identification of the dissection flap. ( **C** ) Left ventricular enlargement and small pleural effusions. ( **D** ) Computed tomography pulmonary angiogram on lung windows showing multifocal ground glass opacification consistent with pulmonary edema.

A 29-year-old female known to have PPCM presented in a state of cardiogenic shock to her local district general hospital 17 weeks of postpartum. Thought to have developed a pulmonary embolism, she underwent a computed tomography pulmonary angiogram ( Fig. 1 ). This demonstrated a Type A aortic dissection. She was referred to a regional aortic center; however, in view of a severe PPCM, they declined, recommending referral to the regional cardiothoracic transplant center for surgery where extracorporeal membrane oxygenation (ECMO) support was available.

Surgery was performed on cardiopulmonary bypass via the right femoral vessels. Both ventricles demonstrated poor function and were dilated ( Video 1 ). A modified Bentall's procedure was performed and, in view of the likely need for postoperative mechanical circulatory support, a tissue aortic valve was used. A composite graft was constructed with a 25-mm Perimount Tissue valve (Edwards Lifescience, Irvine, California) and a 28-mm Gelatin Impregnated Vascular Prosthesis with a 10-mm side arm (Vascutek Terumo, Inchinnan, Renfrewshire, Scotland). The aortic dissection repair was routine with a short period of circulatory arrest and the side arm of the polytetrafluoroethylene (PTFE) graft was used for the arterial cannula of the ECMO circuit.

Following 7 days of postcardiotomy ECMO, with hemodynamic improvement, support was weaned successfully for a period of 3 hours before blood pressure was lost and the patient had to be massaged back onto ECMO. Following an additional 28 days on ECMO, there was further recovery and on this occasion ECMO was weaned successfully ( Video 2 ).

On the 53rd postoperative day, her tracheosotomy was removed and on the 59th day, she was discharged from intensive care.

When she was ultimately seen in clinic, 3 months following discharge, her repeat echo showed nondilated ventricles with only mild impairment of the left and good function of the right.

## Discussion

It is our opinion that the decision to defer this patient's surgery to a center that has regular experience with the use of mechanical circulatory support was crucial to her survival. Many centers in the United Kingdom have experience using postcardiotomy ECMO when they cannot wean patients off cardiopulmonary bypass. However, experience of perfusion teams, ECMO nurses, and intensive care nurses who are proficient in the routine management of these patients is invaluable. 6 We could not find any evidence in the literature, but it seems inevitable that survival of patients having postcardiotomy ECMO in transplant centers will be superior.

Planning in advance that a tissue aortic valve would be used made mechanical circulatory support with ECMO safer. Management of patients with a mechanical aortic valve on ECMO is more challenging when compared with the use of a tissue valve. In such a young patient, the longer term durability of a tissue valve is of concern; however, it was the safer option to bridge the patient through to the short and medium terms. 7

With persisting very poor cardiac function following the aortic dissection repair, consideration was given to listing the patient for transplantation. In view of the residual aortic dissection in the arch and the descending aorta it was decided by the transplant team that she was not a candidate for heart transplantation. In addition, considering the shear stress resulting in the use of a long term implantable left ventricular assist device it was also decided that this would not be an appropriate strategy; this only left the option of trying to bridge to recovery, which was ultimately successful.

This rare presentation of an aortic dissection in the context of PPCM demonstrates an unusual presentation of a lethal condition in a young adult with a weak heart not able to tolerate the stresses of major aortic surgery in this emergency scenario without the use of perioperative mechanical circulatory support. Her recovery required a well thought out strategy and intensive and significant investment of postoperative supportive care.
